# Simultaneous FET-PET and contrast-enhanced MRI based on hybrid PET/MR improves delineation of tumor spatial biodistribution in gliomas: a biopsy validation study

**DOI:** 10.1007/s00259-019-04656-2

**Published:** 2020-01-09

**Authors:** Shuangshuang Song, Ye Cheng, Jie Ma, Leiming Wang, Chengyan Dong, Yukui Wei, Geng Xu, Yang An, Zhigang Qi, Qingtang Lin, Jie Lu

**Affiliations:** 1 Department of Radiology, Xuanwu Hospital, Capital medical University, Beijing, China; 2 Beijing Key Laboratory of Magnetic Resonance Imaging and Brain Informatics, Beijing, China; 3 Department of Neurosurgery, Xuanwu Hospital, Capital Medical University, Beijing, China; 4 Department of Nuclear Medicine, Xuanwu Hospital, Capital Medical University, Beijing, China; 5 Department of Pathology, Xuanwu Hospital, Capital Medical University, Beijing, China; 6 GE Healthcare, Beijing, China

**Keywords:** FET, Contrast-enhanced MRI, Glioma, Tumor volume, Stereotactic biopsy, Hybrid PET/MR

## Abstract

**Purpose:**

Glioma treatment planning requires precise tumor delineation, which is typically performed with contrast-enhanced (CE) MRI. However, CE MRI fails to reflect the entire extent of glioma. O-(2-^18^F-fluoroethyl)-L-tyrosine (^18^F-FET) PET may detect tumor volumes missed by CE MRI. We investigated the clinical value of simultaneous FET-PET and CE MRI in delineating tumor extent before treatment planning. Guided stereotactic biopsy was used to validate the findings.

**Methods:**

Conventional MRI and ^18^F-FET PET were performed simultaneously on a hybrid PET/MR in 33 patients with histopathologically confirmed glioma. Tumor volumes were quantified using a tumor-to-brain ratio ≥ 1.6 (*V*_PET_) and a visual threshold (*V*_CE_). We visually assessed abnormal areas on FLAIR images and calculated Dice’s coefficient (DSC), overlap volume (OV), discrepancy-PET, and discrepancy-CE. Additionally, several stereotactic biopsy samples were taken from “matched” or “mismatched” FET-PET and CE MRI regions.

**Results:**

Among 31 patients (93.94%), FET-PET delineated significantly larger tumor volumes than CE MRI (77.84 ± 51.74 cm^3^ vs. 34.59 ± 27.07 cm^3^, *P* < 0.05). Of the 21 biopsy samples obtained from regions with increased FET uptake, all were histopathologically confirmed as glioma tissue or tumor infiltration, whereas only 13 showed enhancement on CE MRI. Among all patients, the spatial similarity between *V*_PET_ and *V*_CE_ was low (average DSC 0.56 ± 0.22), while the overlap was high (average OV 0.95 ± 0.08). The discrepancy-CE and discrepancy-PET were lower than 10% in 28 and 0 patients, respectively. Eleven patients showed *V*_PET_ partially beyond abnormal signal areas on FLAIR images.

**Conclusion:**

The metabolically active biodistribution of gliomas delineated with FET-PET significantly exceeds tumor volume on CE MRI, and histopathology confirms these findings. Our preliminary results indicate that combining the anatomic and molecular information obtained from conventional MRI and FET-PET would reveal a more accurate glioma extent, which is critical for individualized treatment planning.

**Electronic supplementary material:**

The online version of this article (10.1007/s00259-019-04656-2) contains supplementary material, which is available to authorized users.

## Introduction

For patients with malignant glioma, continuous efforts were made to improve the overall survival and prognosis [1, 2]. Recently, several promising treatment methods, such as the addition of lomustine or tumor-treating fields into standard temozolomide maintenance therapy, shed light on the treatment of malignant glioma, which effectively prolonged 30–55% of median overall survival compared with temozolomide standard therapy [3, 4]. These novel treatments indicated the advancement of the chemotherapies on malignant gliomas and may become new standards of patient care.

As the widely accepted standard treatment for malignant glioma, surgical resection mainly relies on noninvasive imaging to delineate tumor extent. The extent of surgical resection and the residual tumor volumes are pivotal factors that affect the recurrence rate and the prognosis of glioma patients [5, 6]. In general, multimodality imaging plays an essential role in the diagnosis and neurosurgical planning for gliomas [7–10]. Among these approaches, contrast-enhanced (CE) magnetic resonance imaging (MRI) is the mainstay to delineate tumor boundaries and guide further therapies [11]. However, CE MRI, when used alone or in combination with T2 and fluid-attenuated inversion recovery (FLAIR) imaging, may not reliably reflect the entire tumor burden [12, 13]. Consequently, a technique that could delineate the tumor extent more precisely is needed for therapeutic target planning.

The increasing application of positron emission tomography (PET) has improved the diagnosis and clinical management of gliomas [14–18]. The European Association of Nuclear Medicine (EANM), the Society of Nuclear Medicine and Molecular Imaging (SNMMI), the European Association of Neurooncology (EANO), and the working group for Response Assessment in Neurooncology with PET (PET-RANO) have published joint practice guidelines for the use of amino acid PET tracers in glioma imaging and recommended that molecular imaging should be used as a supplement to MRI in the clinical management of gliomas [17]. Compared with 2-deoxy-2-[^18^F]fluoro-d-glucose (^18^F-FDG), amino acid PET tracers, such as ^11^C-methyl-methionine (^11^C-MET), O-(2-^18^F-fluoroethyl)-L-tyrosine (^18^F-FET), and 3,4-dihydroxy-6-[^18^F]fluoro-l-phenylalanine (^18^F-DOPA), exhibit lower uptake in normal brain and inflammatory tissues than in gliomas and thus present clearer tumor borders with a higher tumor-to-background contrast [19, 20]. The half-life of ^18^F (110 min) is longer than that of ^11^C (20 min), making ^18^F-FET more suitable for routine clinical applications in neurooncology [21]. Furthermore, FET has high in vivo stability and is efficiently synthesized by nucleophilic reactions.

Compared with CE MRI, amino acid PET imaging, when used with tracers such as ^11^C-MET [19, 22] and ^18^F-FET [23], often reveals a larger tumor spatial distribution in GBM patients. Among these previous studies, simultaneous PET and MRI acquisition was used with a hybrid PET/MR only in Lohnmann’s study (35 of 50 patients). Other studies used PET and MRI scans obtained on separate occasions, which would therefore not reflect the spatial distribution characteristics of glioma in the same pathological state. In addition, these previous studies were based only on imaging feature assessment without any pathological validation.

In this study, we evaluated the clinical value of hybrid FET-PET/MR in delineating tumor extent and guiding stereotactic biopsies in patients with glioma. The spatial similarity, overlap, discrepancy, and spatial correlation of tumor volumes were analyzed and compared between FET-PET and CE MRI. To better comprehend the imaging findings, several stereotactic biopsy samples were taken from regions showing either substantial overlap or mismatch between FET-PET and CE MR to further assess and validate the clinical potential of hybrid scans in guiding biopsy and surgery.

## Materials and methods

### Patients data

From February 2019 to July 2019, a total of 33 patients with newly diagnosed or recurrent supratentorial gliomas who underwent hybrid ^18^F-FET-PET/MR imaging prior to biopsy or gross resection were retrospectively recruited into this study (Table 1). The diagnosis of glioma was supported by pathological histology according to the 2016 WHO classification system. The Ethics Committee and Institutional Review Board of Xuanwu Hospital Capital Medical University approved this retrospective study and written informed consent was obtained from patients before PET/MR examinations.

**Table 1:** Patient demographics and histopathological characteristics

Characteristic	Data
Total patients	33
Median age, ranges (y)	54.33, 19–73
Sex	
Male	22 (66.67%)
Female	11 (33.33%)
Histologic type	
Astrocytoma	4 (12.12%)
Oligodendroglioma	2 (6.06%)
Glioblastoma multiforme	25 (75.76%)
Ganglioglioma	2 (6.06%)
WHO 2016 grade	
Low grade (grade II)	3 (9.09%)
High grade (grade III–IV)	30 (90.91%)
Newly diagnosed or recurrent	
Newly diagnosed	29 (87.88%)
Recurrent	4 (12.12%)
Tumor-to-brain ratio (TBR)	
Mean	2.35 ± 0.38
Max	4.91 ± 1.33

### Hybrid FET-PET/MR imaging

All PET and MRI examinations were performed simultaneously on a 3-tesla hybrid time-of-flight (TOF) PET/MR (GE Signa, WI, USA) with a 19-channel head-neck coil before biopsy or surgery. A standard MR imaging protocol comprising 3D T2 fluid-attenuated inversion recovery (FLAIR, repetition time/echo time = 9000/maximum), pre- and post-gadolinium 3-dimensional (3D) T1-weighted imaging (T1WI, 8.5/3.2) and axil T1WI, T2WI, FLAIR and diffusion-weighted imaging (DWI) was used. The details of the scan parameters are summarized in Supplementary Table 1.

^18^F-FET was produced and applied as described previously [24]. All patients fasted for at least 4 h before the examination as recommended, and approximately 200 MBq of ^18^F-FET was injected intravenously [17]. After a daily routine emission scan for atlas-based attenuation correction with cylinder source [25], FET-PET acquisition started 20 min after tracer injection and required a total acquisition time of 20 min. PET reconstruction was performed with a TOF-point spread function-ordered subset expectation maximization (TOF-PSF-OSEM, 6 iterations and 16 subsets) algorithm on a 128 × 128 matrix, with a 35 cm axial field-of-view and 2.78 mm slice thickness. Emission data were corrected for scattering, random effects, and dead-time coincidences.

### Postprocessing and comparison of tumor volumes in different modalities

^18^F-FET PET and MR imaging data were postprocessed and analyzed in PMOD version 3.505 (PMOD Ltd.). Different modalities were co-registered using nonaffine deformations and manually adjusted by referring to anatomic landmarks. The static PET images were resliced to the same voxel size as 3D T1 CE MRI with 1 × 1 × 1 mm for robust co-registration and more precise volume calculations of glioma [23].

For tumor volume calculations based on CE MRI (*V*_CE_) data, we used the 3D auto-contouring segmentation method with an individually determined visual threshold judged by two experienced neuroradiologists in consensus (SS Song and ZG Qi) [14, 26]. The value of the lower threshold was adjusted to identify and separate the contrast-enhanced area from nonenhanced brain tissues. For tumor volume calculations based on FET-PET (V_PET_) data, the mean background activity was determined using a merged volume of interest consisting of 3 large crescent-shaped regions in the semi-oval center of the unaffected hemisphere, including white and gray matter [27]. *V*_PET_ was defined by an auto-contouring process with a tumor-to-brain ratio (TBR) threshold of 1.6 or more based on a previous biopsy-controlled study [28]. A manual correction was applied to remove the included blood vessels and other nontumorous tissues with signal intensity or standardized uptake values exceeding the threshold. Necrotic areas and postoperative residual cavities of lesions not showing enhancement or FET uptake were included in the final volume according to surgical criteria [19].

The spatial similarity between *V*_PET_ and *V*_CE_ was determined using Dice’s coefficient (DSC) [29]:

$$ \mathrm{DSC}=2\left(\mid {\mathrm{V}}_{\mathrm{PET}}\cap {\mathrm{V}}_{\mathrm{CE}}\mid \right)/\left(\mid {\mathrm{V}}_{\mathrm{PET}}\mid +\mid {\mathrm{V}}_{\mathrm{CE}}\mid \right). $$

We also calculated the overlap volume (OV) [30], which provides the ratio of the intersection to the smallest volume:

$$ \mathrm{OV}=\left(\mid {\mathrm{V}}_{\mathrm{PET}}\cap {\mathrm{V}}_{\mathrm{CE}}\mid \right)/\min\ \left({\mathrm{V}}_{\mathrm{PET}},{\mathrm{V}}_{\mathrm{CE}}\right). $$

To further evaluate the contributions of FET-PET and CE MRI to delineate tumor volume, we calculated the percentages of discrepancy between volumes obtained by FET-PET and CE MRI. Discrepancy-PET (CE) represents the *V*_PET_ (*V*_CE_) that is not included in *V*_CE_ (*V*_PET_) [19]:

$$ \mathrm{Discrepancy}-\mathrm{PET}=\left(\mid {\mathrm{V}}_{\mathrm{PET}}\cup {\mathrm{V}}_{\mathrm{CE}}\mid -{\mathrm{V}}_{\mathrm{CE}}\right)/\left(\mid {\mathrm{V}}_{\mathrm{PET}}\cup {\mathrm{V}}_{\mathrm{CE}}\mid \right)\times 100\%,\mathrm{and} $$

$$ \mathrm{Discrepancy}-\mathrm{CE}=\left(\mid {\mathrm{V}}_{\mathrm{PET}}\cup {\mathrm{V}}_{\mathrm{CE}}\mid -{\mathrm{V}}_{\mathrm{PET}}\right)/\left(\mid {\mathrm{V}}_{\mathrm{PET}}\cup {\mathrm{V}}_{\mathrm{CE}}\mid \right)\times 100\%. $$

Both the DSC and OV values are between 0 and 1. A Dice coefficient value of 1 indicates perfect agreement between *V*_PET_ and *V*_CE_, and an OV value of 1 indicates that one of the volumes, V_PET_ or V_CE_, completely contains the other. Discrepancy-PET (CE) values range from 0 to 100%. A value of 0 indicates that one of the volumes, *V*_PET_ or *V*_CE_, was completely contained within the other. As the value becomes larger, less of the PET (CE) volume is included in the other.

We next visually assessed the relationship between the abnormal areas on FLAIR and FET-PET images, and divided the 33 patients into 2 types in which *V*_PET_ was either completely included in (type 1) or partially beyond (type 2) the extent of abnormal signal areas shown on FLAIR.

### Stereotactic biopsy procedures

Seven of the patients underwent stereotactic biopsies within a week after PET/MR examinations under the guidance of neuroimaging using co-registered FET-PET and CE MR images loaded into the stereotactic navigation system Robotized Stereotactic Assistant (ROSA, Medtech) [31]. Three to 4 biopsy locations were selected per patient. Biopsy sites were selected only if they were perceived to be safe by both the experienced neurosurgeons (QT Lin and Y Cheng) and neuroradiologists (J Lu and SS Song). Eloquent areas and areas closed to blood vessels were excluded. In all, twenty-four samples were obtained from lesions showing either contrast enhancement and increased FET uptake or increased FET uptake but no enhancement on CE MRI. No postoperative biopsy-related complications were observed. Intraoperative MRI (Siemens, Verio) was used to verify the biopsy site, and 3D Slicer (Version 4.1, SPL, Harvard Medical School) was used to register the preoperative MRI to the intraoperative MRI using the “general registration module.”

All biopsy samples were histologically classified and graded according to the updated 2016 WHO classification of brain tumors [32]. Pathology was determined by two neuropathologists (LM Wang and YS Piao) separately, and discordant results were resolved by consensus. The neuropathologists were blinded to the clinical and imaging data.

### Statistical analysis

Statistical analysis was performed using SPSS, version 22 (IBM). Descriptive statistics are shown as the mean and standard deviation or the median and range. The nonparametric Wilcoxon signed-rank test was applied to compare intergroup differences. The Spearman correlation test was performed to calculate the correlation coefficients of *V*_PET_ and *V*_CE_. *P* values less than 0.05 were considered significant.

## Results

### Tumor volume analysis

**Fig. 1:**
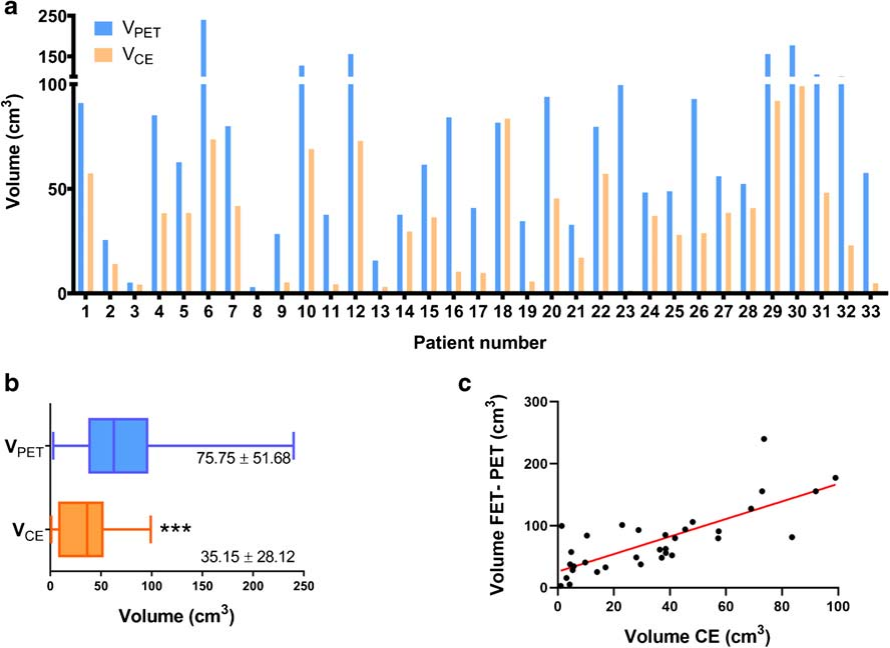
**a***V*_PET_ (yellow) and *V*_CE_ (blue) of the 33 included patients. **b** There was a significant difference in tumor volumes between FET-PET and CE MRI, which produced average values of 75.75 ± 51.68 and 35.15 ± 28.12 cm^3^, respectively. **c** Tumor volume delineated by FET-PET was positively correlated with that delineated by CE MRI (*r* = 0.724). ***P < 0.001

The tumor volume measurements of the 33 patients are shown in Fig. 1a and Supplementary Table 2. The *V*_PET_ and *V*_CE_ of all patients were significantly different (*P* < 0.001, Fig.1b), and the *V*_PET_ and *V*_CE_ were positively correlated (*r* = 0.724, *P* < 0.001, Fig. 1c). *V*_PET_ was significantly larger than *V*_CE_ in 31 patients (93.94%; 77.84 ± 51.74 cm^3^ on FET-PET vs. 34.59 ± 27.07 cm^3^ on CE MRI, *Z* = − 4.860, *P* < 0.001), while *V*_CE_ was larger than *V*_PET_ in only one patient. FET-PET and CE MRI showed similar tumor volumes in one patient (Δ*V* < 1 cm^3^).

### Overlap and discrepancy between *V*_PET_ and *V*_CE_

**Fig. 2:**
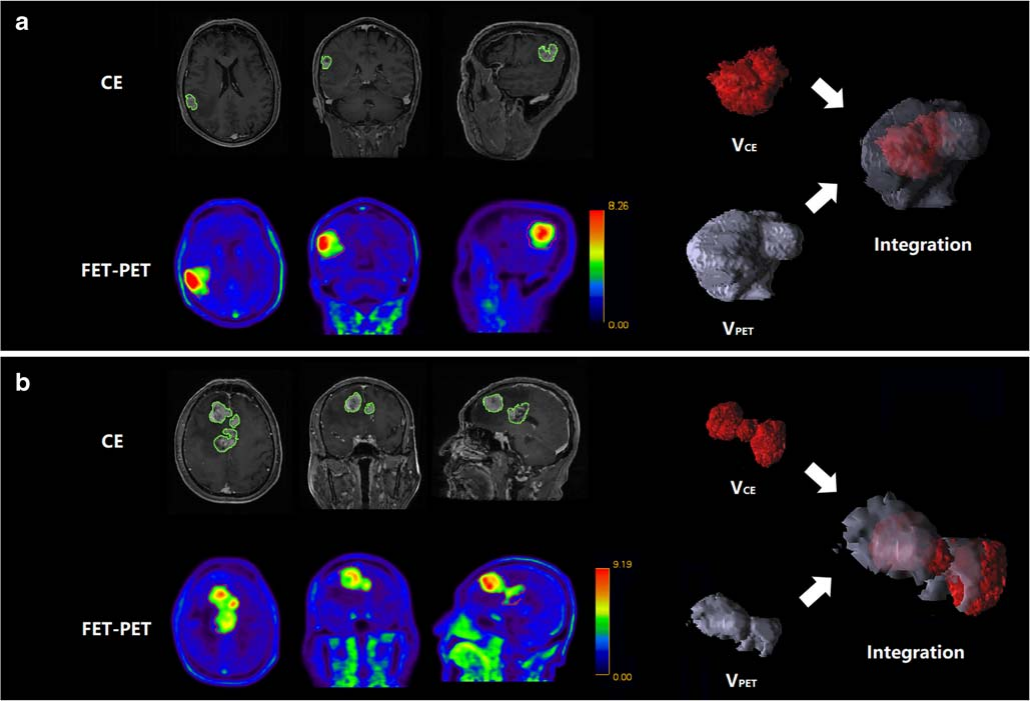
A representative comparison of *V*_CE_ and *V*_PET_ in patients 11 and 27. **a** Patient 11: *V*_PET_ was larger than *V*_CE_ (37.56 vs. 4.39 cm^3^). The discrepancy-CE (0.10%) and DSC (0.21) values were low, while the discrepancy-PET (88.33%) and OV values (0.99) were much higher, indicating that almost all of the *V*_CE_ was contained within *V*_PET_. **b** Patient 27: *V*_PET_ was larger than *V*_CE_ (56.00 vs. 38.52 cm^3^). Both the spatial similarity and overlap were relatively high (DSC 0.53, OV 0.65). However, the discrepancy-PET and discrepancy-CE (44% and 19%, respectively) were both greater than 10%, indicating that FET-PET and CE MRI complemented each other

The average DSC was 0.56 ± 0.22 (range 0.03–0.86). None of the patients had a DSC greater than 0.9, and only 3 patients (9.09%) had a DSC greater than 0.8. Furthermore, eleven patients (33.33%) had a DSC less than 0.5, indicating low spatial similarity between *V*_PET_ and *V*_CE_ (Fig. 2a). In contrast, OV had a larger average of 0.95 ± 0.08 (range 0.65–1.00). Twenty-eight patients (84.85%) had an OV larger than 90% (Fig. 2a and b). In 30 patients (90.91%), *V*_CE_ was almost contained within *V*_PET_, indicated by a discrepancy-CE lower than 10% (mean 1.75 ± 2.23%, range 0–9.23%) (Fig. 2a). None of the patients had a discrepancy-PET less than 10%. However, five patients (15.15%) showed discrepancies between the two modalities of greater than 10% (Fig. 2b). The DSC, OV, discrepancy-PET, and discrepancy-CE metrics of the 33 patients are detailed in Supplementary Table 2.

### The relationship between *V*_PET_ and abnormal areas on FLAIR images

**Fig. 3:**
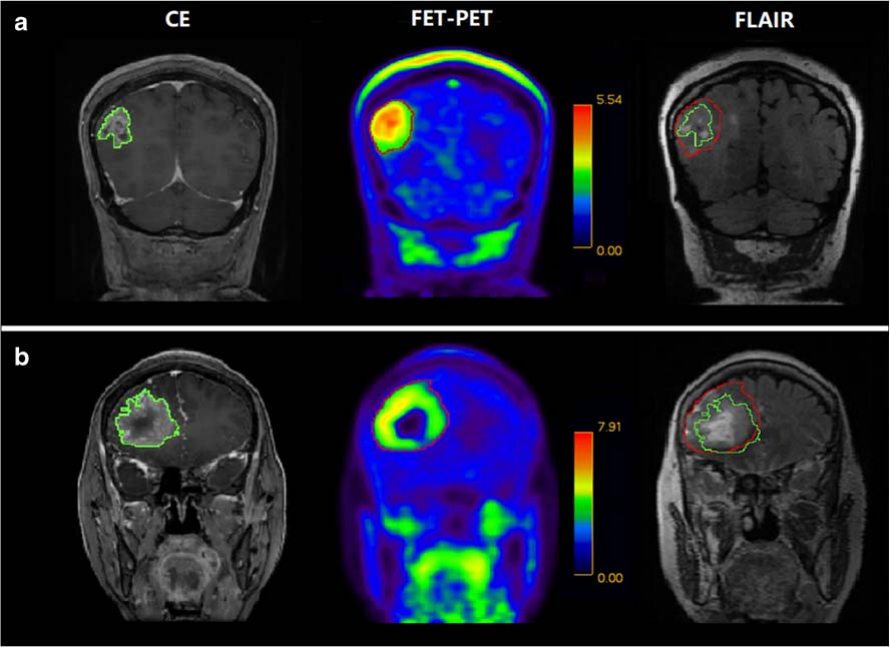
Representative CE MRI, FET-PET images, and the relationships among the abnormal areas identified on CE MRI, FET-PET, and FLAIR images. **a** In patient 19, *V*_PET_ was much larger than *V*_CE_. The abnormal signal areas identified on FLAIR image were larger than both *V*_CE_ and *V*_PET_ (type 1). **b** In patient 29, *V*_CE_ was nearly contained within *V*_PET_. *V*_PET_ was partially beyond the extent of the abnormal signal areas identified on FLAIR image (type 2)

The abnormal signal volumes on FLAIR images were larger than both *V*_CE_ and *V*_PET_. In 22 patients (66.67%), the *V*_PET_ was completely included within the areas of abnormal signal intensity on FLAIR images (type 1, Fig. 3a). In 11 patients (33.33%), *V*_PET_ was partly beyond the areas of abnormal signal intensity on T2 FLAIR (type 2, Fig. 3b).

### Stereotactic biopsy analysis

**Fig. 4:**
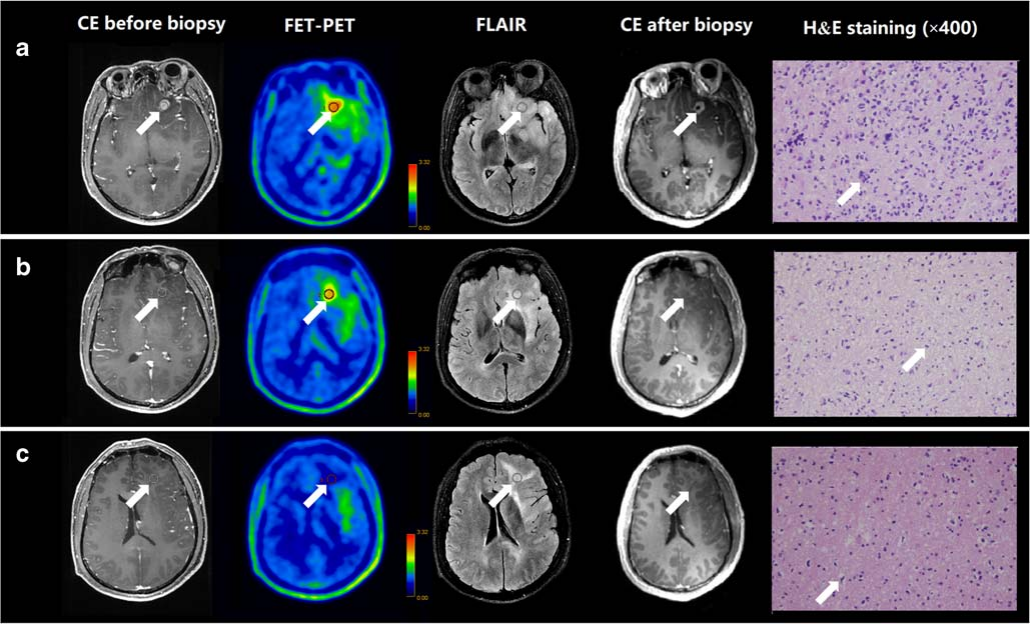
CE MRI, ^18^F-FET-PET and FLAIR performed before biopsy, CE MRI performed after biopsy, and hematoxylin and eosin (H&E) staining (×400) of the biopsy samples. **a** A sample located in the region with increased FET-PET uptake and positive MR enhancement (patient 23). H&E staining showed a cellular glioma corresponding to an astrocytoma of WHO grade II–III with higher local tumor cell density. **b** A sample located in a region with increased FET-PET uptake and negative MR enhancement. H&E staining showed a cellular glioma corresponding to diffuse astrocytoma of WHO grade II. **c** A sample located in a region with abnormal signal areas on FLAIR image that did not show increased FET-PET uptake and contrast enhancement. H&E staining showed this area contained normal brain tissue with a small amount of tumor cell infiltration

A total of 24 samples were obtained from 7 patients by stereotactic biopsy under the guidance of hybrid PET/MR. Thirteen samples were taken from areas with both increased FET uptake and contrast enhancement, 8 samples were obtained from areas with increased FET-PET uptake and no contrast enhancement, and the other 3 samples were obtained from areas negative on both FET-PET and CE MRI but positive on FLAIR images. As expected, all 21 samples obtained from regions of increased FET uptake were pathologically confirmed as glioma tissues or tumor infiltration; however, only 13 of these samples showed contrast enhancement on MRI (Fig. 4). One sample acquired from an area with increased FET uptake but normal signal intensity on FLAIR image was confirmed to contain tumor infiltration by histopathology. Furthermore, three samples were taken from regions with abnormal signal intensity on FLAIR images that were outside of *V*_PET_; and 2 of these 3 samples were confirmed to be normal brain tissue, while one contained a small amount of tumor cell infiltration based on histopathology. The PET/MR imaging features and histopathological results of a total of 24 biopsy samples are listed in Supplementary Table 3.

## Discussion

We found that, in gliomas, a larger proportion of tumor extent was identified by FET-PET than by CE MRI. The histopathology of stereotactic biopsies obtained under the guidance of hybrid FET-PET/MRI confirmed these results. This is the first clinical study to assess tumor volumes simultaneously delineated by FET-PET and MRI using a hybrid PET/MR for gliomas in which the results were validated by stereotactic biopsy. These findings may provide critical information to guide biopsy, surgical, and radiation therapy in patients with gliomas.

During the last few years, interest has increased in the development of applications of ^18^F-FET PET for tumor grading, differential diagnosis, biopsy guidance, and the assessment of treatment response [11, 17, 20, 33]. The amino acid tracer FET may play a more critical role than FDG in the imaging of gliomas because normal brain tissues show a much lower FET uptake; hence, FET provides clearer borders of lesions. Conventional MRI is the primary clinical reference for image-guided surgery. Therefore, it is meaningful to investigate how the data supplied by FET-PET and conventional MRI are different and whether the application of FET-PET shows superiority in gliomas. We found that the contrast-enhanced regions observed on MRI in gliomas were generally contained within *V*_PET_ and there was a positive correlation between *V*_PET_ and *V*_CE_. The mechanisms underlying FET uptake and enhancement on MRI are entirely different. FET is transported by specific stereo-selective amino acid transporters, particularly the large neutral amino acid transporter 1 (LAT1). The transportation of FET is not affected by the blood-brain-barrier [21, 34]. Several recent studies have revealed that areas with increased FET uptake correspond to the tumor cell distribution [16, 35, 36]. However, MR contrast accumulates in gliomas due to blood-brain-barrier disruption and is correlated with the histological features of malignant glioma, including cellularity and proliferation [37]. Additionally, the positive correlation between tumor volumes obtained via FET-PET and CE MRI indicates that some underlying pathophysiological factors may exist. Our results indicate that surgical resection guided by CE MRI alone is inadequate for maximizing patient benefits, whereas FET-PET identifies a greater tumor extent, which is critical for planning therapeutic strategies.

The DSC and OV are different parameters used to evaluate the spatial similarity between tumor volumes on FET-PET and CE MRI, with higher DSC values indicating better consistency between *V*_PET_ and *V*_CE_. The OV value represents the ratio of spatial coincidence volume to the smallest tumor volume. In our study, *V*_CE_ was smaller than *V*_PET_ in 93.94% of the patients. We found that the spatial similarity between FET-PET and CE was low (average DSC, 0.56), while the OV was high (average, 0.95). These findings illustrate that the spatial biodistribution of gliomas is quite different when manifested using FET-PET versus CE MRI and that basing interpretations on the OV alone may be misleading. Combining DSC and OV could produce a more accurate assessment of the spatial similarity between *V*_PET_ and *V*_CE_. Furthermore, *V*_PET_ is larger than *V*_CE_, and *V*_PET_ includes most of *V*_CE_ areas. To validate our findings, we further applied point-to-point stereotactic biopsy studies in 21 regions that showed increased FET uptake with or without MR enhancement. As expected, all samples were confirmed as tumor tissues based on histopathology, but CE MRI identified only 13 (62%) of these regions. Our results support the reliability of FET-PET and the importance of combining both FET-PET and CE MRI to delineate the tumor spatial biodistribution.

To further analyze the differences in glioma spatial biodistribution on FET-PET and CE MRI, we calculated the percentage discrepancy. Two groups of patients with different tendencies were identified. In one group of 30 patients (90.91%), almost all *V*_CE_ areas were included in *V*_PET_, and the discrepancy-CE was less than 10%. However, the other group presented higher discrepancy percentages (> 10%) for both *V*_PET_ and *V*_CE_. These findings are slightly different from those presented in Javier’s study [19],which evaluated the tumor volumes of 23 patients with glioma who preoperatively underwent ^11^C-MET and MRI. In their study, 9 of 23 patients manifested another pattern in which almost all of the tumor volume observed on MET-PET was contained within the MRI (T1 CE MRI or T2WI)-derived volume. However, seven of these 9 patients were pathologically confirmed to have low-grade II gliomas that were negative on contrast enhancement. We recruited only patients with gliomas that were positive on both FET-PET and CE MRI.

Notably, we visually assessed the relationship between tumor extent on FLAIR and FET-PET images and found that 11 patients (33.33%) showed *V*_PET_ areas that partly beyond the abnormal signal area on FLAIR images, in agreement with the study by Lohmann et al. [23]. A similar result was also reported by Grosu et al., who found that areas with higher MET uptake were identified beyond the high-signal areas identified on T2W images in 50% of the patients [38]. These findings indicate that even the combination of CE MRI and FLAIR images is inadequate to delineate the full extent of the tumor in glioma patients. In contrast, Pafundi et al. found that all areas showing increased ^18^F-DOPA uptake were included in the abnormal signal areas on T2W or FLAIR images [12]. In our study, three samples were taken from regions that showed abnormal signal intensity on FLAIR images but were outside *V*_PET_. Based on histopathology, two of these 3 samples were confirmed to be normal brain tissue, but one had a small amount of tumor cell infiltration. This result was consistent with those of Arvizu et al. [19]. In addition, several studies have indicated that tumor volumes based on FLAIR or FET-PET imaging findings are predictive of prognosis in glioma [39–41]. Justin et al. proposed that performing maximum tumor resection according to findings on T2 and FLAIR images was associated with better prognosis in low-grade glioma [41]. Sidsel et al. found that a large tumor volume on FET-PET was an independent predictor of poor survival in GBM patients [42]. The postoperative tumor volume, when based on FET-PET, has also been shown a significant impact on prognosis in GBM patients [39]. FET-PET and FLAIR images show different tumor volumes, and they both play an important role in the evaluating prognosis in glioma; hence, a combination of multimodality imaging to tumor spatial delineation could be valuable for optimal treatment planning.

According to our results, PET/MR is an excellent imaging tool for delineating tumor volumes and identifying glioma boundaries. The integration of PET/MR into the navigation system as well as the development of a preoperative plan are highly recommended. The following limitations should be considered. First, we included a heterogeneous cohort consisting of both newly diagnosed primary and recurrent gliomas in our study. The inclusion of recurrent glioma may have affected the tumor volume assessments because of the effects of previous treatment. However, we did not exclude areas of necrosis or postoperative residual cavities in lesions without enhancement or FET uptake during the tumor volume delineation process according to surgical criteria. Second, no samples were obtained from areas that showed contrast enhancement but no increased FET uptake due to the limited corresponding volumes or their locations (e.g., in eloquence areas or close to blood vessels). Furthermore, a larger cohort of gliomas is needed to confirm the clinical value of multimodality imaging for the management of glioma. Whether FET-PET/MR-guided glioma treatment could improve patient prognosis remains to be investigated in our future work.

In summary, a larger extent of tumor spatial biodistribution was delineated by FET-PET than by CE MRI according to histological confirmation. The spatial similarity between FET-PET and CE MRI was relatively low. Furthermore, our results highlight the importance of combining molecular metabolic imaging (e.g., FET-PET) and anatomic MRI imaging before developing a treatment plan in glioma. Hybrid PET/MR is a promising simultaneous modality that provides opportunities for this application.

## Electronic supplementary material

ESM 1 (DOCX 25 kb)

## References

[1] Stupp R, Hegi ME, Mason WP (2009). Effects of radiotherapy with concomitant and adjuvant temozolomide versus radiotherapy alone on survival in glioblastoma in a randomised phase III study: 5-year analysis of the EORTC-NCIC trial. Lancet Oncol.

[2] Stupp R, Mason WP, van den Bent MJ (2005). Radiotherapy plus concomitant and adjuvant temozolomide for glioblastoma. N Engl J Med.

[3] Herrlinger U, Tzaridis T, Mack F (2019). Lomustine-temozolomide combination therapy versus standard temozolomide therapy in patients with newly diagnosed glioblastoma with methylated MGMT promoter (CeTeG/NOA–09): a randomised, open-label, phase 3 trial. Lancet.

[4] Stupp R, Taillibert S, Kanner A (2017). Effect of tumor-treating fields plus maintenance temozolomide vs maintenance temozolomide alone on survival in patients with glioblastoma. JAMA.

[5] Lacroix M, Abi-Said D, Fourney DR (2001). A multivariate analysis of 416 patients with glioblastoma multiforme: prognosis, extent of resection, and survival. J Neurosurg.

[6] Hervey-Jumper SL, Berger MS (2016). Maximizing safe resection of low- and high-grade glioma. J Neuro-Oncol.

[7] Thust SC, Hassanein S, Bisdas S (2018). Apparent diffusion coefficient for molecular subtyping of non-gadolinium-enhancing WHO grade II/III glioma: volumetric segmentation versus two-dimensional region of interest analysis. Eur Radiol.

[8] Englander ZK, Horenstein CI, Bowden SG (2018). Extent of BOLD vascular dysregulation is greater in diffuse gliomas without isocitrate dehydrogenase 1 R132H mutation. Radiology.

[9] Paech D, Windschuh J, Oberhollenzer J (2018). Assessing the predictability of IDH mutation and MGMT methylation status in glioma patients using relaxation-compensated multipool CEST MRI at 7.0 T. Neuro-Oncology.

[10] Haider SA, Lim S, Kalkanis SN (2019). The impact of 5-aminolevulinic acid on extent of resection in newly diagnosed high grade gliomas: a systematic review and single institutional experience. J Neuro-Oncol.

[11] Albert NL, Weller M, Suchorska B (2016). Response assessment in Neuro-Oncology working group and European Association for Neuro-Oncology recommendations for the clinical use of PET imaging in gliomas. Neuro-Oncology.

[12] Pafundi DH, Laack NN, Youland RS (2013). Biopsy validation of F-18-DOPA PET and biodistribution in gliomas for neurosurgical planning and radiotherapy target delineation: results of a prospective pilot study. Neuro-Oncology.

[13] Pirotte, Benoit JM, Levivier (2009). Positron emission tomography-guided volumetric resection of supratentorial high-grade gliomas: a survival analysis in 66 consecutive patients. Neurosurgery.

[14] Filss CP, Galldiks N, Stoffels G (2014). Comparison of 18F-FET PET and perfusion-weighted MR imaging: a PET/MR imaging hybrid study in patients with brain tumors. J Nucl Med.

[15] Albert NL, Winkelmann I, Suchorska B (2016). Early static 18F-FET-PET scans have a higher accuracy for glioma grading than the standard 20–40 min scans. Eur J Nucl Med Mol Imaging.

[16] Kunz M, Thon N, Eigenbrod S (2011). Hot spots in dynamic (FET)-F-18-PET delineate malignant tumor parts within suspected WHO grade II gliomas. Neuro-Oncology.

[17] Law I, Albert NL, Arbizu J (2018). Joint EANM/EANO/RANO practice guidelines/SNMMI procedure standards for imaging of gliomas using PET with radiolabelled amino acids and [18F]FDG: version 1.0. Eur J Nucl Med Mol Imaging.

[18] Langen K, Watts C (2016). Amino acid PET for brain tumours - ready for the clinic?. Nat Rev Neurol.

[19] Arbizu J, Tejada S, Marti-Climent JM (2012). Quantitative volumetric analysis of gliomas with sequential MRI and 11C-methionine PET assessment: patterns of integration in therapy planning. Eur J Nucl Med Mol Imaging.

[20] Galldiks N, Langen KJ, Holy R (2012). Assessment of treatment response in patients with glioblastoma using O-(2-18F-fluoroethyl)-L-tyrosine PET in comparison to MRI. J Nucl Med.

[21] Langen K, Stoffels G, Filss C (2017). Imaging of amino acid transport in brain tumours: positron emission tomography with O-(2-[ 18 F]fluoroethyl)- L -tyrosine (FET). Methods.

[22] Miwa K (2004). Discrepancy between lesion distributions on methionine PET and MR images in patients with glioblastoma multiforme: insight from a PET and MR fusion image study. J Neurol Neurosurg Psychiatry.

[23] Lohmann P, Stavrinou P, Lipke K (2019). FET PET reveals considerable spatial differences in tumour burden compared to conventional MRI in newly diagnosed glioblastoma. Eur J Nucl Med Mol Imaging.

[24] Hamacher K, Coenen HH (2002). Efficient routine production of the F-18-labelled amino acid O-(2-[F-18]fluoroethyl)-L-tyrosine. Appl Radiat Isotopes.

[25] Ter Voert EEGW, Veit-Haibach P (2017). Clinical evaluation of TOF versus non-TOF on PET artifacts in simultaneous PET/MR: a dual centre experience. Eur J Nucl Med Mol Imaging.

[26] Galldiks N, Ullrich R, Schroeter M (2010). Volumetry of [11C]-methionine PET uptake and MRI contrast enhancement in patients with recurrent glioblastoma multiforme. Eur J Nucl Med Mol Imaging.

[27] Munck Af Rosenschold P, Costa J, Engelholm SA (2015). Impact of [18F]-fluoro-ethyl-tyrosine PET imaging on target definition for radiation therapy of high-grade glioma. Neuro-Oncology.

[28] Pauleit D (2005). O-(2-[18F]fluoroethyl)-L-tyrosine PET combined with MRI improves the diagnostic assessment of cerebral gliomas. Brain.

[29] Henriksen OM, Larsen VA, Muhic A (2016). Simultaneous evaluation of brain tumour metabolism, structure and blood volume using [18F]-fluoroethyltyrosine (FET) PET/MRI: feasibility, agreement and initial experience. Eur J Nucl Med Mol Imaging.

[30] Besemer AE, Titz B, Grudzinski JJ (2017). Impact of PET and MRI threshold-based tumor volume segmentation on patient-specific targeted radionuclide therapy dosimetry using CLR1404. Phys Med Biol.

[31] De Benedictis A, Trezza A, Carai A (2017). Robot-assisted procedures in pediatric neurosurgery. Neurosurg Focus.

[32] Louis DN, Perry A, Reifenberger G (2016). The 2016 world health organization classification of tumors of the central nervous system: a summary. Acta Neuropathol.

[33] Galldiks N, Rapp M, Stoffels G (2013). Response assessment of bevacizumab in patients with recurrent malignant glioma using [18F]Fluoroethyl-l-tyrosine PET in comparison to MRI. Eur J Nucl Med Mol Imaging.

[34] Wester HJ, Herz M, Weber W (1999). Synthesis and radiopharmacology of O-(2-[F-18]fluoroethyl)-L-tyrosine for tumor imaging. J Nucl Med.

[35] Pöpperl G, Kreth FW, Mehrkens JH (2007). FET PET for the evaluation of untreated gliomas: correlation of FET uptake and uptake kinetics with tumour grading. Eur J Nucl Med Mol Imaging.

[36] Jansen N, Graute V, Armbruster L (2012). MRI-suspected low-grade glioma: is there a need to perform dynamic FET PET?. Eur J Nucl Med Mol Imaging.

[37] Ellingson BM, Wen PY, Cloughesy TF (2018). Evidence and context of use for contrast enhancement as a surrogate of disease burden and treatment response in malignant glioma. Neuro-Oncology.

[38] Grosu AL, Weber WA, Riedel E (2005). L-(methyl-11C) methionine positron emission tomography for target delineation in resected high-grade gliomas before radiotherapy. Int J Radiat Oncol.

[39] Piroth MD, Holy R, Pinkawa M (2011). Prognostic impact of postoperative, pre-irradiation 18F-fluoroethyl-l-tyrosine uptake in glioblastoma patients treated with radiochemotherapy. Radiother Oncol.

[40] Suchorska B, Jansen NL, Linn J (2015). Biological tumor volume in 18FET-PET before radiochemotherapy correlates with survival in GBM. Neurology.

[41] Smith JS, Chang EF, Lamborn KR (2008). Role of extent of resection in the long-term outcome of low-grade hemispheric gliomas. J Clin Oncol.

[42] Poulsen SH, Urup T, Grunnet K (2017). The prognostic value of FET PET at radiotherapy planning in newly diagnosed glioblastoma. Eur J Nucl Med Mol Imaging.

