# Supplementary material for: Simultaneous FET-PET and contrast-enhanced MRI based on hybrid PET/MR improves delineation of tumor spatial biodistribution in gliomas: a biopsy validation study
Source: Eur J Nucl Med Mol Imaging. 2020 Jan 9;47(6):1458–67. doi: 10.1007/s00259-019-04656-2 (PMC7188715; doi:10.1007/s00259-019-04656-2)
Supplement: Supplementary file 1 — (DOCX 25 kb) [file 259_2019_4656_MOESM1_ESM.docx]

**Supplementary Materials**

**Supplementary Table 1.** MRI scan parameters

|  | TE  (ms) | TR  (ms) | Thickness/Space (mm) | FOV  (cm) | Matrix | Nex | Slice |
| --- | --- | --- | --- | --- | --- | --- | --- |
| T1WI | 24 | 3002.7 | 5.0/1.0 | 24×24 | 288×256 | 1 | 22 |
| T2WI | 105 | 1806 | 5.0/1.0 | 24×24 | 288×288 | 1 | 22 |
| FLAIR | 145 | 11000 | 5.0/1.0 | 24×24 | 256×256 | 1 | 22 |
| DWI | Minimum | 4675 | 5.0/1.0 | 24×24 | 128×128 | 2 | 44 |
| 3D BRAVO T1 | 3.2 | 8.5 | 1.0/0.0 | 25.6×25.6 | 256×256 | 1 | 188 |
| 3D CUBE FLAIR | Maximum | 9000 | 1.0/0.0 | 25.6×25.6 | 256×256 | 1 | 188 |
| 3D BRAVO T1 Contrast | 3.2 | 8.5 | 1.0/0.0 | 25.6×25.6 | 256×256 | 1 | 188 |

T1WI=T1-weighted imaging; T2WI=T2-weighted imaging; DWI=Diffusion weighted imaging; TE=echo time; TR=repetition time; FOV=field of view; NEX=number of excitations

**Supplementary Table 2.** Patient characteristics, histopathological diagnosis, and tumor volumes information

| NO. | Age | Sex | Location (side) | WHO Grade | Histology | Primary or Recurrence | TBR | | DSC | OV | Discrepancy | | Relation  _PET-FLAIR_ |
| --- | --- | --- | --- | --- | --- | --- | --- | --- | --- | --- | --- | --- | --- |
|  |  |  |  |  |  |  | Mean | Max |  |  | PET (%) | CE (%) |  |
| 1 | 41 | M | P (L) | Ⅳ | GBM | Primary | 2.81 | 0.77 | 0.77 | 0.99 | 37.13 | 0.34 | type 1 |
| 2 | 64 | M | T-I (L) | Ⅳ | GBM | Primary | 2.42 | 0.65 | 0.65 | 0.92 | 47.45 | 4.28 | type 1 |
| 3 | 19 | M | T (R) | Ⅱ | GG | Primary | 1.98 | 0.74 | 0.74 | 0.81 | 28.48 | 13.53 | type 1 |
| 4 | 47 | F | T-O (L) | Ⅳ | GBM | Primary | 2.30 | 0.62 | 0.62 | 0.99 | 55.10 | 0.31 | type 1 |
| 5 | 61 | M | P-T-O (R) | Ⅳ | GBM | Recurrence | 1.95 | 0.73 | 0.73 | 0.96 | 40.09 | 2.25 | type 1 |
| 6 | 44 | M | Fr-P-I (R) | Ⅳ | GBM | Recurrence | 2.65 | 0.47 | 0.47 | 0.99 | 69.37 | 0.11 | type 2 |
| 7 | 48 | M | T (L) | Ⅳ | GBM | Primary | 2.13 | 0.65 | 0.65 | 0.95 | 49.00 | 2.54 | type 1 |
| 8 | 61 | M | Fr (R) | Ⅲ | AA | Primary | 2.66 | 0.50 | 0.50 | 0.96 | 64.84 | 1.41 | type 1 |
| 9 | 68 | M | Fr-I-T (L) | Ⅳ | GBM | Primary | 2.99 | 0.31 | 0.31 | 0.99 | 81.34 | 0.25 | type 1 |
| 10 | 67 | M | H (L) | Ⅳ | GBM | Primary | 2.12 | 0.68 | 0.68 | 0.97 | 46.78 | 1.60 | type 2 |
| 11 | 68 | M | P-T (R) | Ⅳ | GBM | Primary | 2.86 | 0.21 | 0.21 | 0.99 | 88.33 | 0.10 | type 2 |
| 12 | 63 | M | P-O (L) | Ⅳ | GBM | Primary | 1.96 | 0.64 | 0.64 | 1.00 | 53.24 | 0.11 | type 1 |
| 13 | 30 | M | T-I (R) | Ⅱ | GG | Primary | 2.32 | 0.26 | 0.26 | 0.77 | 81.02 | 4.30 | type 2 |
| 14 | 53 | M | Fr-P-T | Ⅳ | GBM | Primary | 2.36 | 0.81 | 0.81 | 0.92 | 26.09 | 6.09 | type 1 |
| 15 | 71 | M | Fr-T-I (L) | Ⅳ | GBM | Primary | 2.40 | 0.72 | 0.72 | 0.97 | 41.89 | 1.76 | type 1 |
| 16 | 37 | M | Fr (R) | Ⅱ | DA | Primary | 3.11 | 0.22 | 0.22 | 0.98 | 87.68 | 0.22 | type 2 |
| 17 | 67 | F | P-O (L) | Ⅳ | GBM | Primary | 2.71 | 0.38 | 0.38 | 1.00 | 76.16 | 0.03 | type 2 |
| 18 | 59 | F | Fr-I-T (R) | Ⅲ | AO | Primary | 2.21 | 0.25 | 0.84 | 0.85 | 12.91 | 14.96 | type 2 |
| 19 | 54 | F | P-O (R) | Ⅳ | GBM | Primary | 2.29 | 0.28 | 0.28 | 1.00 | 83.50 | 0.02 | type 1 |
| 20 | 63 | F | Fr-I-T (L) | Ⅳ | GBM | Primary | 2.15 | 0.65 | 0.65 | 1.00 | 51.68 | 0.02 | type 1 |
| 21 | 61 | F | Fr-I-T (L) | Ⅳ | GBM | Primary | 2.26 | 0.66 | 0.66 | 0.97 | 48.97 | 1.58 | type 1 |
| 22 | 52 | F | Fr (L) | Ⅳ | GBM | Primary | 2.06 | 0.26 | 0.72 | 0.86 | 34.86 | 9.23 | type 2 |
| 23 | 46 | M | Fr-I-T (L) | Ⅲ | AA | Primary | 1.91 | 0.03 | 0.03 | 1.00 | 98.63 | 0.00 | type 2 |
| 24 | 59 | M | P-T (R) | Ⅳ | GBM | Primary | 2.52 | 0.64 | 0.80 | 0.92 | 27.65 | 5.73 | type 1 |
| 25 | 59 | M | P-O (L) | Ⅳ | GBM | Primary | 2.75 | 0.73 | 0.73 | 1.00 | 42.65 | 0.06 | type 1 |
| 26 | 59 | F | H (R) | Ⅳ | GBM | Recurrence | 1.94 | 0.45 | 0.45 | 0.95 | 69.45 | 1.39 | type 1 |
| 27 | 73 | M | Fr (R) | Ⅳ | GBM | Primary | 2.62 | 0.53 | 0.53 | 0.65 | 44.44 | 19.24 | type 1 |
| 28 | 40 | M | Fr-T (L) | Ⅲ | AA | Primary | 1.28 | 0.86 | 0.86 | 0.98 | 23.48 | 1.74 | type 1 |
| 29 | 70 | F | Fr-I (R) | Ⅳ | GBM | Primary | 2.22 | 0.70 | 0.70 | 0.95 | 42.72 | 3.10 | type 2 |
| 30 | 36 | M | Fr (R) | Ⅲ | AO | Primary | 1.91 | 0.68 | 0.68 | 0.95 | 45.51 | 2.46 | type 1 |
| 31 | 56 | M | Fr (R) | Ⅳ | GBM | Primary | 2.47 | 0.61 | 0.61 | 0.97 | 55.22 | 1.31 | type 1 |
| 32 | 67 | F | Fr (L) | Ⅳ | GBM | Primary | 2.47 | 0.37 | 0.37 | 0.99 | 77.34 | 0.23 | type 2 |
| 33 | 30 | F | Fr (L) | Ⅳ | GBM | Recurrence | 2.70 | 0.15 | 0.15 | 1.00 | 91.66 | 0.00 | type 1 |

NO. patient number, M male, F female, P parietal, T temporal, I insular, O occipital, Fr frontal, H hemisphere, L left, R right, GBM glioblastoma multiforme, GG ganglioglioma, AA anaplastic astrocytoma, DA diffuse astrocytoma, AO anaplastic oligodendroglioma, Relation_PET-FLAIR_ relation of tumor areas between FET-PET and FLAIR

**Supplementary Table 3.** A summary of the stereotactic biopsy results of each sample

| Patient number | Imaging features of sample | | | histopathology | WHO grading |
| --- | --- | --- | --- | --- | --- |
|  | Contrast enhancement | FLAIR | FET-PET |  |  |
| 4 | Negative | abnormal | Positive | AA | Ⅲ |
| 4 | Positive | abnormal | Positive | GBM | Ⅳ |
| 4 | Positive | abnormal | Positive | AA | Ⅲ |
| 11 | Positive | abnormal | Positive | GBM | Ⅳ |
| 11 | Negative | abnormal | Negative | No tumor | - |
| 11 | Negative | normal | Positive | Infiltrative tumor | - |
| 11 | Negative | abnormal | Positive | GBM | Ⅳ |
| 14 | Positive | abnormal | Positive | GBM | Ⅳ |
| 14 | Positive | abnormal | Positive | GBM | Ⅳ |
| 14 | Negative | abnormal | Positive | Infiltrative tumor | - |
| 14 | Negative | abnormal | Negative | No tumor | - |
| 20 | Positive | abnormal | Positive | DA | Ⅱ |
| 20 | Negative | abnormal | Positive | Infiltrative tumor | - |
| 20 | Negative | abnormal | Positive | Infiltrative tumor | - |
| 20 | Positive | abnormal | Positive | DA | Ⅱ |
| 23 | Positive | abnormal | Positive | DA-AA | Ⅱ-Ⅲ |
| 23 | Positive | abnormal | Positive | DA | Ⅱ |
| 23 | Negative | abnormal | Negative | Infiltrative tumor | - |
| 31 | Positive | abnormal | Positive | GBM | Ⅳ |
| 31 | Positive | abnormal | Positive | GBM | Ⅳ |
| 31 | Negative | abnormal | Positive | AA | Ⅲ |
| 32 | Positive | abnormal | Positive | AA | Ⅲ |
| 32 | Positive | abnormal | Positive | GBM | Ⅳ |
| 32 | Negative | abnormal | Positive | DA | Ⅱ |

GBM glioblastoma multiforme, AA anaplastic astrocytoma, DA diffuse astrocytoma, infiltrative tumor, normal brain tissue with a small amount of infiltrative tumor cells
